# Incentive EMR Sharing System Based on Consortium Blockchain and IPFS

**DOI:** 10.3390/healthcare10101840

**Published:** 2022-09-22

**Authors:** Wanbing Zhan, Chin-Ling Chen, Wei Weng, Woei-Jiunn Tsaur, Zi-Yi Lim, Yong-Yuan Deng

**Affiliations:** 1 School of Computer and Information Engineering, Xiamen University of Technology, Xiamen 361024, China; 2 School of Information Engineering, Changchun Sci-Tech University, Changchun 130600, China; 3 Department of Computer Science and Information Engineering, Chaoyang University of Technology, Taichung City 413310, Taiwan; 4 Computer Center, National Taipei University, New Taipei City 237303, Taiwan; 5 Department of Computer Science and Information Engineering, National Taipei University, New Taipei City 237303, Taiwan; 6 Department of Information and Communication Engineering, Chaoyang University of Technology, 168 Jifeng East Road, Taichung 413310, Taiwan

**Keywords:** EMR sharing, consortium blockchain, access control, proxy re-encryption, IPFS

## Abstract

Electronic medical records (EMRs) are extremely private data in the medical industry. Clinicians use the patient data that the EMR stores to quickly assess a patient’s status and save diagnostic information. In the conventional medical model, it is easy for duplicate exams, medical resource waste, or the loss of medical records to happen when a patient is transferred between several medical facilities due to problems with data sharing and exchange, inadequate data privacy, security, confidentiality, and difficulties with data traceability. This paper recommends a Hyperledger Fabric-based strategy to promote the exchange of EMR models. With the use of Hyperledger Fabric, EMR stakeholders can be brought into the channel to facilitate data sharing. Attribute-based access control (ABAC) allows users to design the data access control policy, and the data access control may improve security. Any record stored in the blockchain can be viewed using the Hyperledger Fabric feature and it cannot be altered or destroyed, ensuring data traceability. Through proxy re-encryption, which makes sure that the data is not leaked during data exchange, data secrecy can be ensured. A module for medical tokens has now been added. Many foreign medical institutions currently use the medical token system, and the system described in this paper can use the tokens to pay for some medical expenses. The tokens are obtained by the patient’s initiative to share their EMR with the medical institution for research, which is how many foreign medical institutions currently use the medical token mechanism. This paradigm can encourage the growth of medical data by enabling stakeholders to collaborate and share EMR trust.

## 1. Introduction

### 1.1. Background

Digital patient medical records are preserved, transmitted, and managed using electronic devices, which are referred to as “electronic medical records.” The value of the current electronic medical records (EMRs) increased by about $31 billion globally from 2000 to 2018 due to the expansion of the internet and information technology [1]. The growth of EMRs is attributable to the fact that it has comprehensive patient medical records and offers hospitals and patients many conveniences [2]. A survey found that just 32% of provincial hospitals had built electronic medical record systems for usage, despite the rising adoption of EMRs worldwide [3].

The data in the traditional model of the system is stored in a centralized way, making it vulnerable to numerous dangers, including human manipulation and hacking, which can result in data loss [4,5]. At the moment, EMRs have apparent flaws in storage security and data exchange. At the same time, there is a lack of data sharing and difficulty in exchanging medical data between various medical institutions, which casts doubt on the process of data sharing, makes it easy for patients to repeat exams and raises costs. As significant patient personal information is held in EMRs, blockchain technology can be utilized in conjunction with EMRs to create strong data shareability and data security. This will help to alleviate some of the problems with the existing EMR paradigm. The InterPlanetary File System (IPFS) is introduced as the storage medium for medical data and the data in the chain is stored in the hash address returned by IPFS to achieve decentralized data storage, because the volume of media data in medical data is not suitable to all be deposited in the chain [6].

Bitcoin, the first virtual currency, was conceptualized and created by Satoshi Nakamoto in January 2009. A distributed database made up of several nodes in a peer-to-peer (P2P) network is used by the Bitcoin economy to confirm and record all transactions, and a cryptographic design is used to assure the security of all aspects of currency circulation [7]. Based on the fact that, as of the current listing, more than 23 different healthcare coins (health-based cryptocurrencies) have been exchanged, medical digital currencies provide better convenience than the conventional legal tender used for transactions [8]. A virtual wallet can be used and accessed from anywhere on the globe as long as there is an internet connection. One of the more representative ones is the SOLVE token from the Solve.care platform, which is dedicated to facilitating coordination, management, and payment in the healthcare process [9]. The Medibloc token serves as the Medibloc group’s group token, Medipass is mostly used for mobile phones, and Dr. Palette is utilized by patients to manage their EMR, while Panacea was created based on a blockchain kernel to store sensitive user data [10].

Medipass is mostly used for mobile phones. To allow different parties in the network to communicate through the Doc.com token, including, but not limited to leveraging Doc.com for healthcare support, the academy has developed a blockchain platform [11].

In this article, we suggest the use of the medical cryptocurrency Medcoin to encourage patients to switch from the conventional EMR sharing model to a blockchain-based medical data sharing model, which can effectively address both the problem of data security and the problem of data sharing. In this work, we show how utilizing Hyperledger Fabric and proxy re-encryption can improve the security of EMR.

The use of proxy re-encryption permits data to be stored in ciphertext for the duration of its storage lifespan [12]; the proxy service provider, however, is unable to access the data in plaintext since it lacks access to the private keys of the involved communicating parties. The proxy service provider need not be completely trusted for the participating communication parties to have sufficient security to store the data. The threat model that this paper’s suggested system can successfully thwart is as follows.

1. Data integrity [13]: This term refers to data accuracy and dependability. To verify the integrity of the original medical data, peer-to-peer data transmission using Hyperledger Fabric channels can provide good protection against network attacks and prevent the leakage of sensitive information;
2. Unauthorized access [14]: In a setting with multiple users, the system administrator denies the user access to system files or the system itself, and the user instead accesses resources secretly. To accomplish identity verification authorization, Hyperledger Fabric’s Membership Service Provider (MSP) identity management reads the X.509g format ID book; to accomplish access authorization to system resources, ABAC uses attribute-based access control;
3. Human forgery and data tampering problem [15]: In the conventional paradigm, due to centralized storage of medical data, tampering or forgery of private medical data can seriously jeopardize the safety of patients’ lives if there are untrusted nodes or attacks by malevolent nodes in the network. Therefore, the original data can be protected by the tamper-proof and traceable characteristics of blockchain. To prevent data fabrication and manipulation, the medical data can be signed using the Elliptic Curve Digital Signature Algorithm (ECDSA) signature method and then the message digest algorithm can produce the hash;
4. Collusion attack issue [16]: The proxy re-encryption method is used to safeguard the authorized party’s data security to prevent unauthorized third-party organizations from leaking the patient’s private key, which would allow the user’s encrypted data to be decrypted. It enables the authorized party to use its private key to decrypt the required material without disclosing its private key [17].

### 1.2. Related Works

Today’s common solutions in the field of EMR research center on leveraging blockchain technology to store patient EMRs and encryption to guard against unwanted data manipulation. Since most research takes the form of public chains, it is challenging to address the issue of resource access given the chain’s finite amount of storage. The relevant works are listed in Table 1, although there are still some issues with them.

Xia et al. proposed a blockchain-based data sharing framework that is based on a permissioned blockchain [18]. The chain allows only invited and therefore authenticated users to access it, while each access is recorded in the transaction history and kept by the blockchain. The traditional model of data control and sharing dilemma is solved, but the process of the ID book signature algorithm is not clearly described, and the access control module lacks flexibility in the custom access policy model part.

Wang et al. proposed a decentralized scheme based on secret policy attribute encryption (CP-ABE) combined with the Ethernet public chain for access control [19]. The behavior of accessing data parties is monitored and recorded in the chain, and access control policies can be created through the access policy tree to achieve more flexible access control compared to the traditional model. However, at the same time, it increases the storage burden in the chain and the burden on the data owner.

Alsalamah et al. developed Wholesome Coin to encourage people to exercise in order to promote healthy living programs [20]. Wearable internet devices are used to monitor physical exercise data and coins are issued through blockchain technology. Coins are used as the core of an incentive mechanism to encourage people to adhere to the exercise program, and coins can be used to pay for medical services. However, this move is prone to malicious coin number swiping and coin abuse by dishonest parties.

Mani et al. proposed a patient-centered EMR management with IPFS as a medium for storing medical data; the hash address of their IPFS is stored on the chain, which solves the problem of blockchain data storage [21]. However, sufficient security measures are not taken for the data in IPFS and there is still a risk of data leakage.

Kan et al. proposed a proxy re-encryption scheme of CAA-secure to solve the problem of decentralized storage security, which is able to free chosen ciphertext attacks [22]. This scheme reduces the cost of replicating proxy keys in a distributed environment and only one set of key pairs is required to prevent collusion attacks.

However, to prevent confidential information leakage, the keys need to be replaced periodically. Therefore, the research objectives of the EMR sharing system proposed in this paper are as follows: to provide a sharing method for EMR data that combines the federated chain’s tamper-proof and traceable properties with the security authorization capabilities of ABAC and proxy re-encryption. The suggested solution stores EMRs behind an IPFS chain, and proxy re-encryption is used to secure data sharing authorization. In order to enable diverse data access capabilities for users and devices with varied attributes, the transaction information is also maintained in the federated chain and the information of various users is written into digital certificates. To distribute EMR data using federated chain technology, create medical currency using blockchain technology, and create a reward system between the two, Medcoin is introduced.

**Table 1:** Comparison between the proposed and existing enterprise data sharing solutions.

Authors	Year	Technologies	Object	Merits	Demerits
Xia et al. [18]	2017	Blockchain	Solve the problem of data shareability and control in the cloud through blockchain.	Leverages the concept of shared request pools to share data and enhances the security of data in the cloud through secure encryption technology.	The mathematical formula process for detailed secure encryption is not specified.
Wang et al. [19]	2019	Ethereum CP-ABE	Access control of data in cloud storage is achieved through Ethernet combined with secret attribute policy-based encryption.	A decentralized system is realized, and the resource management module is delivered to smart contracts for processing, which can effectively prevent the third-party platform from being vulnerable to attacks.	This solution increases the storage burden of the data owner to a certain extent to ensure data security. It is difficult to afford in the chain in case of excessive volume.
Alsalamah et al. [20].	2021	WIOT Blockchain Wholesome Coin	Use IoT devices to monitor people’s physical health data, issue medical currencies through blockchain technology, and create a reward mechanism between the two.	Secure circulation of coins is ensured through smart contracts.	Wholesome Coin is at risk of misuse and the access control model is vulnerable to human vulnerability.
Mani et al. [21].	2021	Hyperledger Fabric IPFS	Using IPFS to store medical data to solve the blockchain storage capacity problem.	Blockchain technology is used to solve the poor shareability of medical privacy data by adopting an on-chain and off-chain dual storage model to solve the storage capacity problem.	The data stored in IPFS is not encrypted using cryptographic techniques and the level of data security is not high enough.
Kan et al. [22].	2020	Proxy Re-Encryption Blockchain	Reduce the cost of re-encryption key replication in a decentralized storage environment with CAA-secure’s proxy re-encryption solution.	Simplifies its re-encryption key process compared to collusion proxy re-encryption and is collision-resistant.	Since there is only one set of key pairs for encryption and decryption, the keys need to be changed periodically to prevent collusion attacks, which causes inconvenience.

The following organizational scheme guides the writing of this essay: The key technologies used in the system of this paper are introduced in Section 2; the architecture and main operating procedures of the system are described in Section 3; a security analysis is conducted in Section 4; the computational cost, communication performance, and comparison are covered in Section 5; and our proposal is wrapped up in Section 6.

## 2. Preliminary

### 2.1. Hyperledger Fabric

Utilizing Hyperledger Fabric technology, the federated blockchain network includes the following features: fine-grained access control based on authentication; pluggable consensus methods for subscription services; and distributed and chained storage of transaction records [23].

**Figure 1:**
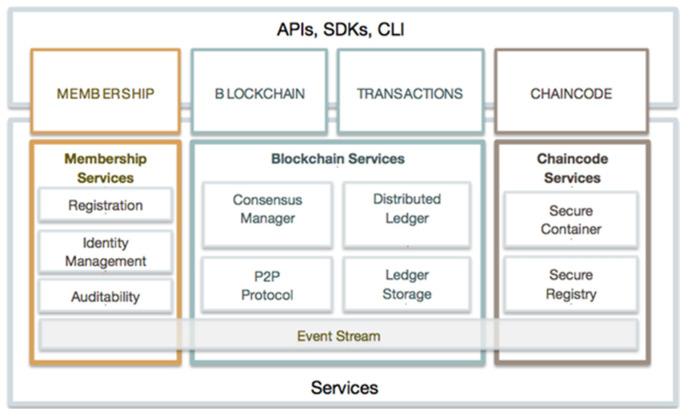
Hyperledger Fabric architecture.

A blockchain implementation platform called Hyperledger Fabric offers a flexible, scalable architecture and plug-and-play compatibility for its components (such as consensus algorithms). Additionally, it makes use of container technology to house smart contracts, often known as “chain code”, which make up the system’s application logic. The Go and JavaScript chain code, as well as other languages like Java, are supported by the Hyperledger architecture [24]. Therefore, compared to the Ethereum blockchain network, which only supports a closed smart contract language known as solid, the Hyperledger architecture is more adaptable. Developers can also access a development toolset, which offers a practical development foundation. Figure 1 depicts one of the Hyperledger Fabric frameworks.

PKI, decentralized/consensus technology, and member services transform blockchains without permissions into blockchains with permissions. Members must register with an entity to receive enrollment certificates, which may be generated based on the type of entity, and must be licensed before they may join the network. The distributed ledger is managed by the blockchain service using a peer-to-peer (peer-to-peer) protocol through HTTP/2. In order to offer the most effective hashing technique to maintain the replication of the world state, the data structure is highly optimized. In each deployment, various consensus algorithms (PBFT, Raft, PoW, PoS) can be added and customized. The Smart Contract of Fabric A, also known as “chaincode”, manages the business logic decided upon by the network’s participants. System configuration, user transaction signatures, and verification techniques are all implemented using the system chaincode. The init method and the invoke method of the system chaincode are executed by the node process. When the chaincode receives an initialization request, the init method is invoked, and the invoke function takes and processes various transaction request offers. When the requirements for calling chaincode are satisfied by the transaction request, Hyperledger Fabric will automatically launch and communicate with the ledger. “Blockchain” and “state” are the two main blocks in the ledger. A chain of interconnected blocks, called a “blockchain”, is used to store past transactions. For the server side chaincode, Fabric offers APIs that make application development easier. Currently, Fabric supports development in Go, Java, or Node.js. The Node.js and Java SDKs are presently available from Fabric for client-side applications.

### 2.2. InterPlanetary File System (IPFS)

Data that has been encrypted is kept on IPFS. IPFS is a distributed, decentralized file system [25]. The idea behind it is to replace domain-based addresses with content-based addresses, which means that consumers should check for content instead of addresses. Each file in IPFS is given a distinct hash value. Data access is accelerated and made more safe, robust, and long-lasting via IPFS, which searches files based on the hash value. 

### 2.3. Proxy Re-Encryption

The best strategy to prevent data privacy leaks in this article is to employ the user’s public key encryption to secure the security of the data; the user just has to preserve his private key. This is because the EMR needs to be kept in IPFS through the cloud service provider. The encrypted files cannot be opened by the hospital because patients must share the EMR when they visit, and the straightforward public–private key encryption can only be broken by the patients themselves. Therefore, secure and effective proxy re-encryption is required to transform the cipher text so that the applicant may easily decrypt the patient’s encrypted data. Currently, distributed file systems and access control both make extensive use of PRE technology.

In elliptic curve-based proxy re-encryption, let E be an elliptic curve over a limited field $F_{q}$, where q is a large prime number and G is a point on the elliptic curve E of order n [26]. Let G1, G2 be two multiplicative cyclic groups of prime modulo n. Let e: G1×G1→G2 be a bilinear map z=e(G1,G2)∈G2 [27].

Let the elliptic curve equation f represent the information embedding function, which maps information M to a point $P_{m}$ on E [28], with the data owner and B as the requesting party.

Key generation phase: let a private key $d_{A}\in Z_{n}^{*}$ and a public key $Q_{A}=d_{A}G$.

Encryption plaintext phase: generate an arbitrary number $r\in Z_{n}^{*}$ and output the ciphertext $\left(C_{1},C_{2})=(rQ_{A},z^{r}G+P_{m}\right)$.

Re-encryption key generation phase: user A authorizes user B to decrypt data using its private key. The re-encryption key is $rk_{A\to B}=d_{A}^{-1}Q_{B}$.

Re-encryption process phase: use the re-encryption key to encrypt the ciphertext twice:

$$\begin{array}{l} \left(C_{1}^{\prime },C_{2}^{\prime }\right)\\ =(e(rQ_{A},rk_{A\to B}),z^{r}G+P_{m})\\ =(e(rQ_{A},d_{A}^{-1}d_{B}G),z^{r}G+P_{m})\\ =(z^{rd_{B}},z^{r}G+P_{m}) \end{array}$$

Ciphertext decryption phase: B can decrypt the $P_{m}$ with key $d_{B}$:

$$P_{m}=C_{2}^{\prime }-{\left(C_{1}^{\prime }\right)}^{\frac{1}{d_{B}}}G$$

Then, apply the inverse of the function f to obtain the original message m from $P_{m}$.

### 2.4. Attribute-Based Access Control (ABAC)

**Figure 2:**
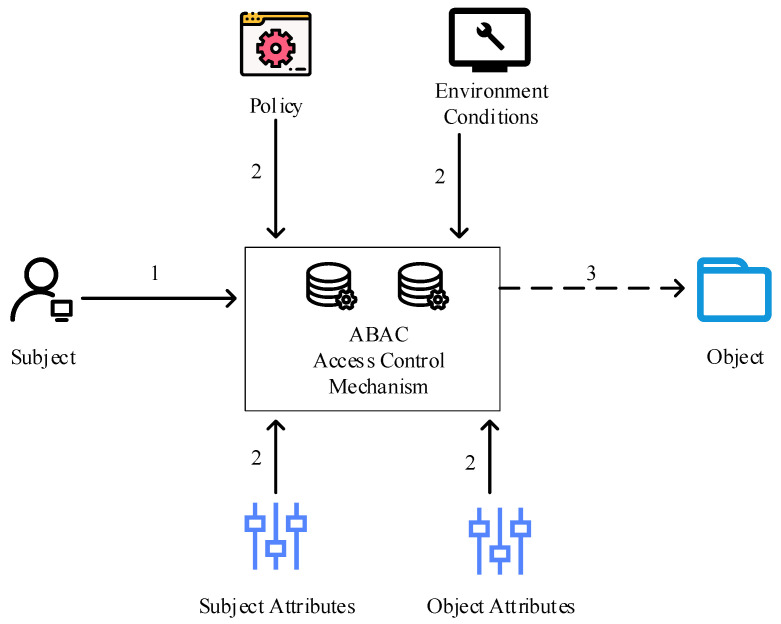
Attribute-based access control model.

In order to establish fine-grained access control in this work, ABAC is primarily utilized to evaluate and calculate subject attributes (Subject), object attributes (Object), environmental conditions (Environment), and access control rules (Policy) or policies [29]. The final strategy is the object that serves the output. The following benefits of ABAC include its ability to accomplish various levels of access control granularity in accordance with various requirements, its cheap access control management costs, and its ability to create flexible and dynamic overall control. The attribute-based access control model is depicted in Figure 2 below.

### 2.5. Elliptic Curve Digital Signature Algorithm (ECDSA)

In 2000, the IEEE and NIST adopted the elliptic curve digital signature technique as a standard [30]. Unlike integer factorization problem IFP, the elliptic curve discrete logarithm problem ECDLP lacks a sub-exponential time solution. Smaller parameters, shorter keys, shorter signatures, and faster computation time are benefits of ECDLP. With the help of this capability, ECDSA may be used with Hyperledger Fabric, partially resolving the issue of scarce processing and storage resources.

Suppose role A signs the message m as a signer, role B verifies the message legitimacy as a verifier, and A chooses elliptic curve parameters as $y^{2}=(x^{3}+ax+b)mod\;p$, the base p, and the origin G. This creates the key pair $\left(d_{A},Q_{A}\right)$ of role A: the private key $d_{A}$ and the public key $Q_{A}=d_{A}G$.

The signature process of A is as follows:

1. Role A selects an elliptic curve $E_{p}(a,b)$ and a base point G;
2. Role A chooses a random number k∈[1,N−1]; N is the order of G;
3. Role A calculates the information hash H=hash(m);
4. Role A calculates a point (x,y)=kG;
5. Role A calculates r=xmod n, r≠0; $s=k^{-1}(H+rd_{A})mod\;n$; (r,s) is the ECDSA signature result of role A, which is sent to role B.

The verification process for B is as follows:

1. Role B computes the hash of m, $H^{\prime }=hash(m)$;
2. Role B calculates $u_{1}=s^{-1}H^{\prime }mod\;n$, $u_{2}=s^{-1}rmod\;n$;
3. Role B calculates $(x^{\prime },y^{\prime })=u_{1}G+u_{2}Q_{A}$;
4. If $x^{\prime }=r$, then signature verification is successful.

## 3. System Overview

### 3.1. System Architecture

**Figure 3:**
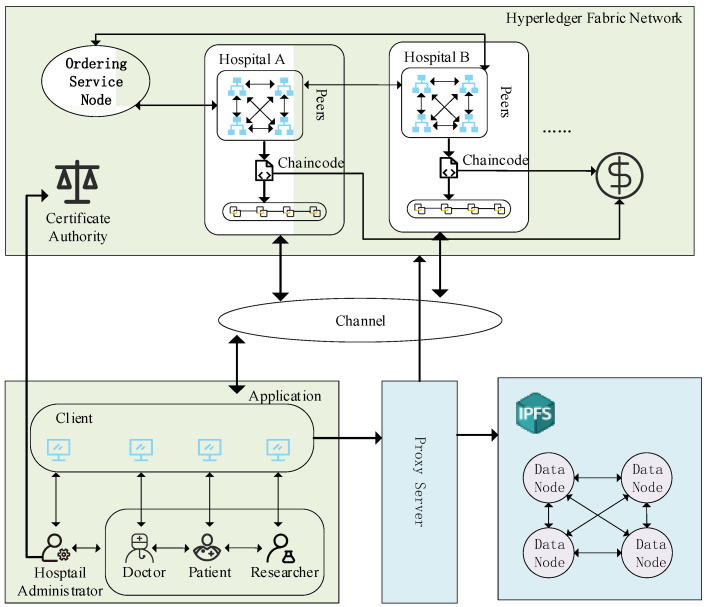
The consortium blockchain service center architecture.

In this research, we propose an architecture for a consortium blockchain-based system, as seen in Figure 3. The consortium blockchain network module, the application and service module, and the data storage module make up the system architecture’s three main modules.

1. Consortium Blockchain Center (CBC)

The Consortium Blockchain Center is a distributed network service made up of many healthcare organizations that offer consumers distributed data storage and blockchain services (such as token issuance, data query, and data on-chain). The CBC can only be joined by and used by authorized nodes. The access method allows CBC to select a more adaptable consensus mechanism, significantly increasing the effectiveness of data access and storage.

2. Application layer and service layer

The application gives patients, doctors, researchers, and hospital administrators access to EMR system services. Through the service layer API made available by the HTTP server, the client communicates with the IPFS storage and the backend blockchain network. The service layer secures the keys during data transmission and serves as middleware for proxy re-encryption operations. The service layer separates the application and data layers in this manner.

3. Data layer

The data layer consists of two parts: IPFS and the consortium blockchain. The traditional blockchain system sacrifices “efficiency” for “security”, and the capacity and rate of stored data are relatively low, which is not suitable for storing large-scale data. Based on this consideration, we use blockchain (Hyperledger Fabric) and distributed storage (IPFS) to solve the problem of large-scale data chains. The patient’s encrypted medical records are stored in the IPFS distributed system with addresses linked to the blockchain. Users can access this data through the address information of the encrypted medical records on the blockchain. Furthermore, to ensure that the data on IPFS is not tampered with, the fingerprints (hashes) of the encrypted medical records must be stored in the blockchain, which verifies the integrity and reliability of the data.

### 3.2. Application Scenario

There are five entities in the overall application scenario.

Consortium blockchain center (CBC):

Multiple hospital groups with distributed storage capabilities make up the distributed web service known as the CBC. The CBC primarily uses digital ID certificate issuance, user identity management, digital signature verification, transaction archiving, and transaction processing. When patients and doctors engage with the Hyperledger Fabric network, their signatures are checked by the alliance chain network and when the transaction is legal, the matching response is implemented using chaincode.

Hospital administrator (HA):

HA is in charge of managing the hospital organization and patient payments in the hospital organization that makes up the blockchain center. It is also in charge of luring members and organizations into the channel. Through the chain code, HA is recorded in the blockchain center.

Doctor (D) and Researcher (R):

Users who are doctors and researchers must register with the CBC through CA. The patient’s previous medical records may be requested by the doctors and researchers for purposes of diagnosis and academic study. Once the patient has been diagnosed, the doctor will create an encrypted medical record using the patient’s public key and link the record’s data using a chain code.

Patient (P):

The chain code is used to register the patient with the CBC. Patients are holders of their EMRs and patients use the Hyperledger Fabric network to share EMRs with their stakeholders, that is, doctors. Doctors can read their historical EMRs with permission thanks to configurable access policies specified via certificate characteristics. In addition, hospital users can permit researchers to see their past medical records. In addition to storing EMRs in IPFS, the patient also records the returned hash as chain transaction metadata.

**Figure 4:**
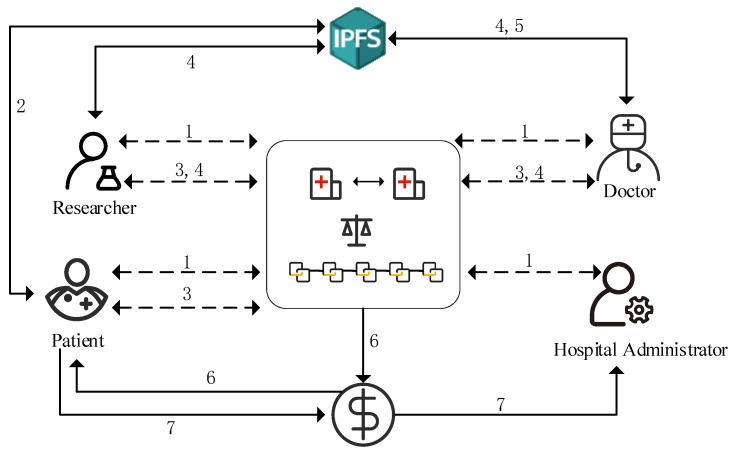
The system framework.

The specific interaction process of the system is shown in Figure 4.

Step 1: Doctors, patients, researchers, and hospital administrators register with the Fabric Certificate Authority (CA) through the client in the Hyperledger Fabric blockchain network. The CA receives the registration application and generates the public key, private key, and certificate for each role (insert the field with the attribute “ROLE” in the certificate for attribute-based access control), and sends them to the corresponding role;

Step 2: The patient encrypts his or her EMR with the received public key, uploads it to IPFS for storage, and IPFS returns the hash of the EMR address. The patient uploads the personal information and the returned hash address as transaction information and signs it;

Step 3: The doctor or researcher initiates a request to the patient to view his EMR, and the patient can authorize the operation according to the attribute-based access control, while the patient returns the transaction information to the chain after using the applicant’s public key for the proxy re-encryption operation;

Step 4: After receiving the transaction information from the patient, the application data party obtains the EMR encrypted data located in the IPFS through its hash address. Since the patient has performed proxy re-encryption, he/she can decrypt the encrypted data with his/her private key;

Step 5: After the diagnosis is completed, the doctor can modify the EMR, sign it, and store it in IPFS;

Step 6: If the patient actively shares the EMR with the hospital, the default is to share it with their researcher, and Hyperledger Fabric issues medical tokens (MedCoin);

Step 7: Patients can obtain MedCoin as an incentive to use MedCoin to offset part of their medical expenses through the hospital administrator after the diagnosis is completed.

### 3.3. Initial Phase

**Figure 5:**
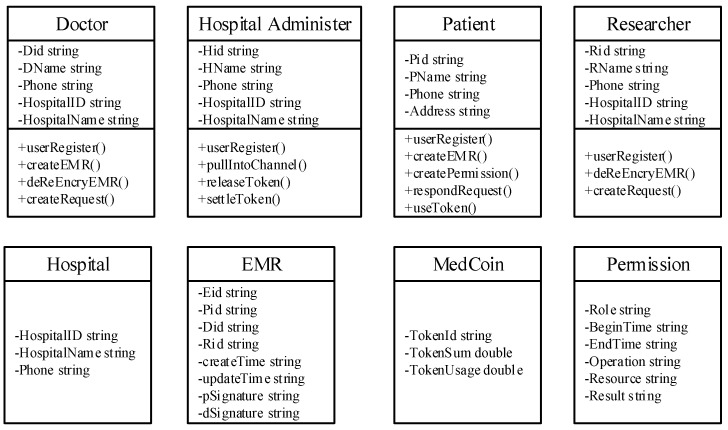
The data structure.

In the initialization phase, we set up an extensible federated blockchain network in accordance with the Hyperledger Fabric structure. Thanks to the careful planning of the data structure, the definition of the corresponding chain code, and some algorithms, this network can effectively implement the business logic of the fundamental medical record system. The data structure design is shown in Figure 5.

### 3.4. Registration Phase

**Figure 6:**
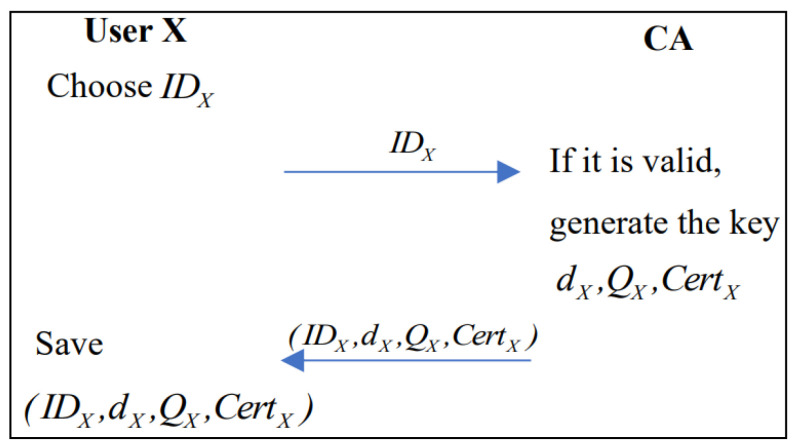
The data structure.

In the registration phase, for identities not registered in the Fabric CA (X) all nodes including patients, doctors, researchers, and hospital administrators that want to join the Hyperledger Fabric network need to interact with the CA to issue digital certificates. We use ′Users X′ to represent all arbitrary roles in the blockchain system. Figure 6 shows the flowchart of the registration phase.

Step 1. The user submits the registration information $ID_{X}$ through the client ($ID_{X}$ is the registration information of all the roles participating in the system) and sends it to the CA node in the CBC for registration through the client;

Step 2. The CA generates the private key $d_{X}$ and calculates the public key $Q_{X}=d_{X}G$ according to ECDSA, with G as the base point. If the registration information of user X is legitimate, the CA returns the signed private key $d_{X}$, public key $Q_{X}$, and certificate $Cert_{X}$ of registered user X. The $Cert_{X}$ contains the ′Role′ attribute, which is used to identify the identity;

Step 3. The user saves $\left(ID_{X},d_{X},Q_{X},Cert_{X}\right)$.

### 3.5. Data Storage Phase

**Figure 7:**
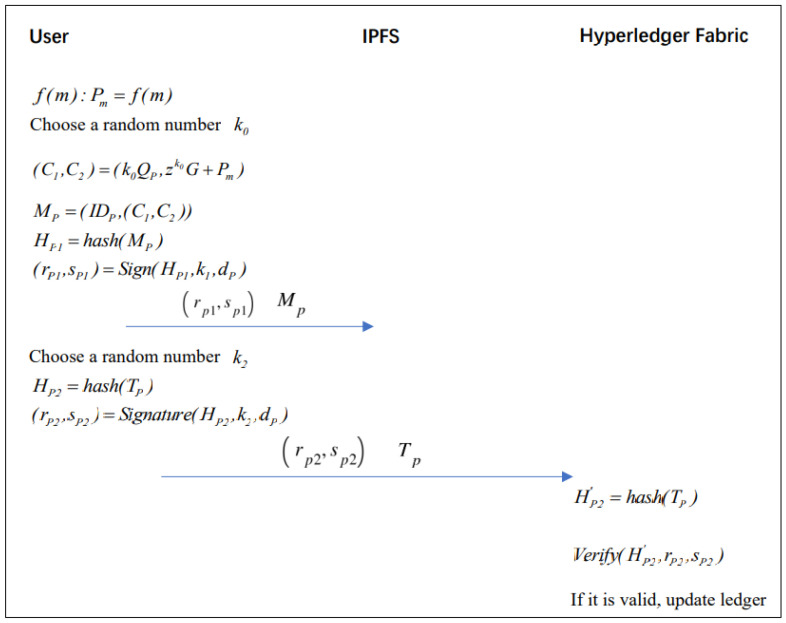
Data storage phase flow chart.

The two main processes in the data storage phase are, first, storing the EMR in IPFS, and second, storing the transactional data in the blockchain network. The data storage phase flow chart in Figure 7 displays the overall flow chart.

#### 3.5.1. EMR Storage in IPFS

We follow the call of the related algorithm until the fourth chapter, and its specific code is placed in the Appendix A.

If the patient is using the proposed system for the first time, the EMR needs to be encrypted and signed by the client, and then uploaded to IPFS with the following detailed process.

Step 1: Patients encrypt their medical records by calling the encrypted algorithm in Algorithm A1 (Please see the Appendix A). Choose a random number $k_{0}$ to encrypt the medical record data m=data and obtain the ciphertext $\left(C_{1},C_{2}\right)$.

$$\begin{array}{c} f(m):P_{m}=f(m)\\ (C_{1},C_{2})=(k_{0}Q_{P},z^{k_{0}}G+P_{m}); \end{array}$$

Step 2: Patients randomly select a random number $k_{1}$ and patient EMR information $M_{P}$

$$M_{P}=(ID_{P},(C_{1},C_{2}))$$

The patient generates a signature $\left(r_{P1},s_{P1}\right)$ by calling the Signature function of the ECDSA signature algorithm and selecting a random number $k_{1}$. The signature function is shown in Algorithm A2 (Please see the Appendix A).

$$H_{P1}=hash(M_{P})\;(r_{P1},s_{P1})=Sign(H_{P1},k_{1},d_{P});$$

Step 3: The patient stores the signature $\left(r_{P1},s_{P1}\right)$ in IPFS with the patient medical record information $M_{P}$ and IPFS returns the hash value of the data address.

#### 3.5.2. Transaction Storage in Chain

Step 1: The patient user writes the hash address returned by IPFS into the transaction $T_{P}$ (the transaction information can be used as data credentials) and calls the ECDSA algorithm Signature function to generate a signature $\left(r_{P2},s_{P2}\right)$. After signing the transaction, it is submitted to the Hyperledger Fabric network.

Signed: The patient randomly selects a random number $k_{2}$ and the transaction information $T_{P}$

$$H_{P2}=hash(T_{P})\;(r_{P2},s_{P2})=Sign(H_{P2},k_{2},d_{P});$$

Step 2: Verify the nodes in the network through the verify function of Algorithm A3 (Please see the Appendix A). If the verification is passed, it is submitted to the blockchain network by the sorting node.

Verify: The nodes in the network calculate the hash of the transaction information

$$H_{P2}^{\prime }=hash(T_{P})\;Verify(H_{P2}^{\prime },r_{P2},s_{P2})$$

**Figure 8:**
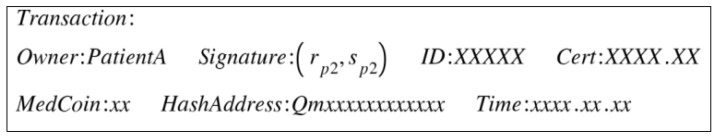
Data storage phase flow chart.

The transaction information submitted by the user to the blockchain network is shown in the transaction information structure in Figure 8.

### 3.6. Attribute-Based Access Control

Through the client, patients can create unique attribute-based access controls. Their unique attributes are represented by a key-value pair with arbitrary values for both the key and the value. When various roles are registered, the attributes are included in the certificates that Fabric CA issues. The organization to which the identity belongs is defined by Affiliation, Type identifies the type of identity, and EnrollmentID specifies the ID of the registered identity (for example, a doctor belongs to Hospital A).

Subject, Object, Operation, and Environment are the four components of attribute-based access control, with Attribute serving as the attribute of the four. Together, these components provide user-defined access control policies.

Attribute∈{Su,Ob,Op,En}

The policy establishes the access rule and either allows or rejects access requests depending on whether all access requests comply with the rule.

Policy(Su,Ob,Op,En})→{allow,refuse}

In this study, the hash of the medical history address recorded in the chain is the object, which relates to the resources handled by ABAC. In this study, the term “operation” refers to the tasks that the subject must complete, including reading the chain’s resources. The environment in this document is used to set the policy’s effective time and expiration time. The environment is a term that refers to the contextual activities for the current application access request. In this work, the effective time and expiration time of the policy are set using the context operation of the present application access request, or “Environment”.

Environment=(BeginTime,EndTime)

The Subject attribute requires the insertion of ‘ROLE’ attributes in the certificate, and the insertion of custom attributes in the digital certificate can be achieved according to Algorithm A4 (Please see the Appendix A).

Step 1: The patient defines the access policy format. Algorithm A5 (Please see the Appendix A) is the pseudo-code implementation to define the access policy. The operation item describes the type of operation that can be performed on the accessed resource. The resource item describes the resource object to be operated on, and the subject item describes the object of the access policy;

Step 2: The strategy is set as follows.

$$Policy(doctor||researcher\wedge T_{P}\wedge read||update\wedge BeginTime||EndTime)\to \mathrm{allow}$$

If all access control conditions are met, the output allow is allowed.

### 3.7. Hospital Request for Data Access Phase

When a patient arrives at the hospital, the doctor submits a request to access the patient’s electronic medical record. To validate the request’s validity, the starting actor must call the Signature function of Algorithm A2 and sign the request. By using the Verify function of Algorithm A3 to validate the signature, the Hyperledger Fabric network does so. By executing the Verify function of Algorithm A3 and verifying the signature, the Hyperledger Fabric network first confirms that the request complies with the patient’s access rules.

Step 1: Signature: The doctor randomly selects a random number $k_{3}$ to generate the request message

$$Req_{D}(ID_{D},Cert_{D},operation,object,timestamp)\;H_{D1}=hash(Req_{D})\;\left(r_{D1},s_{D1})=Sign(H_{D1},k_{3},d_{D}\right)$$

To verify the signature, the nodes in the network calculate the hash of the request message

$$H_{D1}^{\prime }=hash(Req_{D})\;Verify(H_{D1}^{\prime },r_{D1},s_{D1})$$

According to the attribute values of the access policy, the Hyperledger network reads the ROLE value in the certificate, operation, object, and timestamp in the request.

Policy(Su,Ob,Op,En)

If it matches, “allow” is output, which means it is accessible.

Policy(Su,Ob,Op,En)→allow;

Step 2: The patient sets the re-encryption key by reading the public key from the applicant’s certificate in the request message after receiving the request from the applicant and passing the tailored access policy.

$$rk_{A\to B}=d_{P}^{-1}Q_{D}=d_{P}^{-1}d_{D}G$$

The proxy server calls Algorithm A6 (Please see the Appendix A) to re-encrypt the EMR in IPFS in order to protect the patient’s privacy from leakage and chooses a random number *k*_4_ to obtain the re-encrypted ciphertext $\left(C_{1}^{\prime },C_{2}^{\prime }\right)$

$$\begin{array}{l} \left(C_{1}^{\prime },C_{2}^{\prime }\right)\\ =(e(k_{4}Q_{P},rk_{A\to B}),z^{k_{4}}G+P_{m})\\ =(e(k_{4}Q_{P},d_{P}^{-1}d_{D}G),z^{k_{4}}G+P_{m})\\ =(z^{k_{4}d_{D}},z^{k_{4}}G+P_{m}) \end{array}$$

The patient stores the re-encrypted ciphertext $\left(C_{1}^{\prime },C_{2}^{\prime }\right)$ in IPFS and also returns the transaction information to the applicant chain;

Step 3: After the applicant receives the authentication message, he or she obtains the hash address through the transaction information to find the corresponding EMR data in IPFS. Since the data owner sets the proxy re-encryption key, calling Algorithm A7 (Please see the Appendix A) allows the applicant to decrypt the EMR with his or her private key.

$$P_{m}=C_{2}^{\prime }-{\left(C_{1}^{\prime }\right)}^{\frac{1}{d_{D}}}G$$

Finally, the obtained data is inserted and removed as the original EMR of the patient.

$$m=f^{-1}(P_{m}).$$

### 3.8. Diagnosis Phase

After the physician’s diagnosis is complete, the patient EMR can be updated, encrypted, and signed using the patient’s public key and then deposited into IPFS with the following process.

Step 1: The doctor selects a random number $k_{5}$ to encrypt the updated medical record m1=data1 by calling Algorithm A1 to obtain the ciphertext $\left(C_{D1},C_{D2}\right)$

$$f(m1):P_{m1}=f(m1)\;(C_{D1},C_{D2})=(k_{5}Q_{P},z^{k_{5}}G+P_{m1});$$

Step 2: The doctor randomly selects a random number $k_{6}$ and updates the medical record information $M_{P1}$

$$M_{P1}=(ID_{D},(C_{D1},C_{D2}))$$

The doctor generates the signature $\left(r_{D2},s_{D2}\right)$ by calling the Signature function of the ECDSA signature algorithm of Algorithm A2.

$$H_{D2}=hash(M_{P1})\;(r_{D2},s_{D2})=Sign(H_{D2},k_{6},d_{D});$$

Step 3: The Hyperledger Fabric network verifies the signature by calling the Verify function of Algorithm A3.

$$H_{D2}^{\prime }=hash(M_{P1})\;Verify(H_{D2}^{\prime },r_{D2},s_{D2})$$

If the verification passes, the physician stores the signature $\left(r_{D2},s_{D2}\right)$ in the IPFS with the patient’s medical record information $M_{P1}$.

### 3.9. Token Generation Phase

We suggest a “Sharing-Reward” strategy to encourage individuals to share their medical records more, which will make it easier for researchers to evaluate medical records and locate pathology. The patient will receive a set percentage of the token reward if he or she agrees to submit their medical data when the doctor or researcher requests to examine it. First, in the proposed system, we develop a token currency called MEDCOIN. The patient can purchase medications from the hospital or pay for diagnostic services using MEDCOIN tokens. The “data island” situation brought on by data encryption is resolved using the “Sharing-Reward” concept and the proxy re-encryption technique. The token Algorithm A8 (Please see the Appendix A) and the “Sharing-Reward” model are depicted here.

## 4. Analysis

### 4.1. Mutual Authentication

In the system scenario proposed in this paper, we use BAN Logic to authenticate both sides of the data transfer. The BAN Logic syntax notation and semantics are as follows.

P|≡X P believes X is real

P⊲X P receives a message containing X

P|∼X P has sent a message containing X

P|⇒X P has jurisdiction over X

$P\overset{K}{↔}Q$ K is a shared key between P and Q

$\overset{K}{\to }Q$ K is used as the public key of P

#(X) fresh(X), X is the latest

${\left\{X\right\}}_{K}$ X is encrypted by key K

${\langle X\rangle }_{Y}$ X combined with Y

In this phase, both communicating parties are authenticated by BAN Logic. Taking user A and user B as an example, A as a physician and B as a patient, the main purpose is as follows.

$$G1:A|\equiv A\overset{K_{B-A}}{↔}B$$

$$G2:A|\equiv B|\equiv A\overset{K_{B-A}}{↔}B$$

$$G3:B|\equiv A\overset{K_{A-B}}{↔}B$$

$$G4:B|\equiv A|\equiv A\overset{K_{A-B}}{↔}B$$

$$G5:A|\equiv ID_{B}$$

$$G6:A|\equiv B|\equiv ID_{B}$$

$$G7:B|\equiv ID_{A}$$

$$G8:B|\equiv A|\equiv ID_{A}$$

$$G9:A|\equiv rk_{B-A}$$

$$G10:A|\equiv B|\equiv rk_{B-A}$$

The following idealized form is generated by BAN Logic:

$$M1:A\to B({\left\{ID_{A},k_{3},Req\right\}}_{Q_{B}},{\langle h(ID_{A},k_{3},Req)\rangle }_{K_{A-B}})$$

$$M2:B\to A({\left\{ID_{B},k_{4},rk_{A\to B},Req\right\}}_{Q_{A}},{\langle h(ID_{B},k_{4},rk_{A\to B},Req)\rangle }_{K_{A-B}})$$

The analysis scheme is carried out based on the following assumptions:

$$A1:B|\equiv \#(k_{3})$$

$$A2:A|\equiv \#(k_{4})$$

$$A3:A|\equiv B|\Rightarrow A\overset{K_{A-B}}{↔}B$$

$$A4:B|\equiv A|\Rightarrow A\overset{K_{A-B}}{↔}B$$

$$A5:A|\equiv B|\equiv ID_{B}$$

$$A6:A|\equiv B|\equiv ID_{A}$$

$$A7:A|\equiv B|\Rightarrow rk_{A\to B}$$

$$A8:A|\equiv \overset{Q_{B}}{\to }B$$

$$A9:B|\equiv \overset{Q_{A}}{\to }A$$

The verification hypothesis is proven as follows.

User B authenticates user A:

Through M1 and the seeing rule, we can derive:

(1) $$B⊲({\left\{ID_{A},k_{3},Req\right\}}_{Q_{B}},{\langle h(ID_{A},k_{3},Req)\rangle }_{K_{A-B}})$$

Through A2 and the freshness rule, we can derive:

(2) $$B|\equiv \#({\left\{ID_{A},k_{3},Req\right\}}_{Q_{B}},{\langle h(ID_{A},k_{3},Req)\rangle }_{K_{A-B}})$$

Through Equation (1), A9, and the message meaning rule, we can derive:

(3) $$B|\equiv A|∼({\left\{ID_{A},k_{3},Req\right\}}_{Q_{B}},{\langle h(ID_{A},k_{3},Req)\rangle }_{K_{A-B}})$$

Through Equations (2) and (3), and the nonce verification rule, we can derive:

(4) $$B|\equiv A|\equiv ({\left\{ID_{A},k_{3},Req\right\}}_{Q_{B}},{\langle h(ID_{A},k_{3},Req)\rangle }_{K_{A-B}})$$

Through Equation (4) and the belief rule, we can derive (G4):

(5) $$B|\equiv A|\equiv A\overset{K_{A-B}}{↔}B$$

(6) $$B|\equiv A|\equiv ID_{A}$$

Through Equation (5), A4, and the jurisdiction rule, we can derive (G3):

(7) $$B|\equiv A\overset{K_{A-B}}{↔}B$$

Through Equation (5), A6, and the jurisdiction rule, we can derive (G7):

(8) $$B|\equiv ID_{A}$$

User A authenticates user B:

Through M2 and the seeing rule, we can derive:

(9) $$A⊲({\left\{ID_{B},k_{4},rk_{A\to B},Req\right\}}_{Q_{A}},{\langle h(ID_{B},k_{4},rk_{A\to B},Req)\rangle }_{K_{A-B}})$$

Through A2 and the freshness rule, we can derive:

(10) $$A|\equiv \#({\left\{ID_{B},k_{4},rk_{A\to B},Req\right\}}_{Q_{A}},{\langle h(ID_{B},k_{4},rk_{A\to B},Req)\rangle }_{K_{A-B}})$$

Through Equation (9) and the message meaning rule, we can derive:

(11) $$A|\equiv B∼({\left\{ID_{B},k_{4},rk_{A\to B},Req\right\}}_{Q_{A}},{\langle h(ID_{B},k_{4},rk_{A\to B},Req)\rangle }_{K_{A-B}})$$

Through Equations (10) and (11), and the nonce verification rule, we can derive:

(12) $$A|\equiv B|\equiv ({\left\{ID_{B},k_{4},rk_{A\to B},Req\right\}}_{Q_{A}},{\langle h(ID_{B},k_{4},rk_{A\to B},Req)\rangle }_{K_{A-B}})$$

Through Equation (12) and the belief rule, we can derive (G2), (G6), and (G10):

(13) $$A|\equiv B|\equiv A\overset{K_{B-A}}{↔}B$$

(14) $$A|\equiv B|\equiv ID_{B}$$

(15) $$A|\equiv B|\equiv rk_{A\to B}$$

Through Equation (13), A3, and the jurisdiction rule, we can derive (G1):

(16) $$A|\equiv A\overset{K_{B-A}}{↔}B$$

Through Equation (14), A5, and the jurisdiction rule, we can derive (G5):

(17) $$A|\equiv ID_{B}$$

Through Equation (15), A7, and the jurisdiction rule, we can derive (G9):

(18) $$A|\equiv B|\equiv rk_{A\to B}$$

Equations (7), (8), (16) and (17) show that mutual authentication between roles A and B is possible.

### 4.2. Data Integrity

In the method suggested in this research, we employ the elliptic curve digital signature technique ECDSA for signing and verifying the message on both ends of the communication, which is used to assure the reliability of the data source. The data owner also encrypts the EMR using his or her public key, and only his or her private key can be used to decrypt the ciphertext, protecting the integrity of the data. This work employs the proxy re-encryption approach to protect the privacy of the data owner’s private key from being compromised and to enable the accessing party to decrypt the ciphertext using its private key in order to assure data security.

Taking the data access phase as an example, when a physician wants to request a view of a patient’s EMR, he or she needs to calculate its hash for the requested message and sign it using his or her signature $\left(r_{D1},s_{D1}\right)$ to send it to the patient. The message is received by the patient and verified by the verification algorithm in the ECDSA calculation.

$$\begin{array}{l} H_{D1}^{\prime }=hash(Req_{D})\\ u_{1}=s_{D1}^{-1}H_{D1}^{\prime }mod\;n\\ u_{2}=s_{D1}^{-1}r_{D1}mod\;n\\ (x_{D1}^{\prime },y_{D1}^{\prime })=u_{1}G+u_{2}G \end{array}$$

The data integrity can be effectively guaranteed by the ECDSA method if the validation succeeds, which indicates that the application is legitimate, and fails, which indicates that the message has been tampered with or a fake message has been sent by a hacker attack. Once the verification has been successful, the patient can hash, sign, and send the chain of data to the doctor. Since the data is kept in a chain structure, changing the hash value of the entire chain would be required to alter the data at this point, which is not possible in the current computer environment.

### 4.3. Traceability

All transactions made by the participants in the federated chain are recorded as transactions placed in the chain and cannot be maliciously altered because the blockchain is employed as the fundamental idea. The system can establish good traceability and eliminate fraud at its source because the records are permanent.

Scenario: The patient has a sudden exacerbation, etc.

Analysis: The signature verification method in the interaction process can ensure that it can be precisely traced to the consulting physician for fast treatment by utilizing the tamper-evident and traceable features of blockchain. For example, before a physician needs to view a patient’s EMR stored in IPFS, he or she needs to initiate a request to the patient. To ensure the authenticity of the individual’s identity, the doctor needs to sign $\left(r_{D1},s_{D1})=Sign(H_{D1},k_{3},d_{D}\right)$, the application information, before the data is uploaded to the chain, and the signature will be automatically verified by Hyperledger Fabric. We can verify the signature of the role in the previous operation phase; the verification equation is

$$\begin{array}{l} u_{1}=s_{D1}^{-1}H_{D1}^{\prime }mod\;n\\ u_{2}=s_{D1}^{-1}r_{D1}mod\;n\\ (x_{D1}^{\prime },y_{D1}^{\prime })=u_{1}G+u_{2}G \end{array}$$

If $x^{\prime }=r$, it means the data is legal.

### 4.4. Non-Repudiation

Every stage of the technique that is suggested in this work uses ECDSA to provide non-repudiation. The receiver checks the signature after receiving the message and each role in the system must sign communications and store data using its private key. The message can be delivered automatically once the recipient has confirmed its validity and authenticity. The non-repudiation of the roles at each level is displayed in Table 2.

In this paper, we adopt attribute-based access control (ABAC), which gives the data owner the flexibility to customize the access control policy, and the system reads the ROLE attributes from the visitor’s credentials. The data owner develops the policy as follows.

$$Access\;Policy=(doctor||researcher\wedge T_{P}\wedge read||update\wedge BeginTime||EndTime)$$

1. Access role: The ROLE attribute in the certificate is required to be in this set; the attribute of the patient is ‘Patient’, the attribute of the doctor is ‘Doctor’, and the attribute of the researcher is ‘Researcher’.

2. Access time duration: Access must only be permitted during this period, and no one else may access the data at any other time.

3. Data operations: This collection must include both the operation attributes and the operations that users can execute on the data, such as adding, deleting, and altering it. Since the patient’s public key encrypts the EMR in IPFS, only the patient’s private key can be used to decrypt the original data if he or she needs to access it. However, the patient’s private key can be effectively protected from leakage by setting the re-encryption key through the proxy re-encryption service when the applicant interacts with the patient. The re-encryption key setting formula is $rk_{A\to B}=d_{A}^{-1}Q_{B}$.

### 4.5. Known Attacks

#### 4.5.1. Man-in-the-Middle Attack

Scenario: For example, during communication between a patient and a doctor, an attacker intercepts the request message sent by the doctor or intercepts the data transmitted by the patient and sends it to the destination after maliciously tampering with it.

Analysis: In the protocol outlined in this work, public key encryption is used and both communicating parties compute their hash before sending data $\left(C_{1},C_{2})=(rQ_{A},z^{r}G+P_{m}\right)$. Man-in-the-middle attacks are effectively prevented because, if an attacker tampers with the data on purpose, it is impossible to decode the data, and the receiver will discover this right away while checking the hash.

#### 4.5.2. Replay Attack

Scenario: The attacker intercepts the communication between the two parties while the data is being transmitted, resends the message to the recipient by pretending to be a legal message, and attempts to trick the recipient into responding with the necessary data.

Analysis: The system in this paper allows patients to customize the access policy, where the policy includes the access time.

Access time∈Check(EndTime−BeginTime)

The patient will only be given permission once within this time. The access period is created and allowed using ABAC when the patient receives the requested information from the doctor for the first time and the verification is successful. The random number k is used in each communication at a different value, such as $k_{2}$ in the patient transaction information upload phase and *k*_3_ in the doctor request access data phase.

$$\left(r_{P2},s_{P2})=Sign(H_{P2},k_{2},d_{P}\right)\;\left(r_{D1},s_{D1})=Sign(H_{D1},k_{3},d_{D}\right)$$

Each operation will be signed with a random number of different values in the signature, so replay attacks are not feasible in this scheme.

#### 4.5.3. Collusion Attack

Scenario: Hospital doctors collude with proxy service providers to obtain patients’ private keys.

Analysis: In the proposed scheme, the patient’s private key is protected by proxy re-encryption, which is naturally resistant to collusion. After the patient receives the request information from the doctor, the re-encryption key is calculated using the inverse of the doctor’s public key $Q_{D}$ in the request and his or her private key $rk_{A\to B}=d_{P}^{-1}Q_{D}$.

In this process, neither the doctor nor the proxy service provider has direct access to the patient’s private key unless the patient actively discloses his or her private key.

## 5. Performance Evaluation

### 5.1. Communication Cost

The following table shows the communication analysis of the proposed scenario in this paper. In the 4G network environment, the maximum transmission speed is 100Mbps, and in the 5G network environment, the maximum transmission speed is 20 Gps. The ECDSA signature has a length of 160 bits, the key has a length of 160 bits, the hash function has a length of 256 bits, the transaction information length is 1024 bits, the request information length is 1024 bits, and other information (such as identification ID, etc.) is 80 bits in length.

Let us take the data access phase with the highest communication cost as an example. First, the doctor needs to send a request to the patient, which includes a signature massage, a hash, a request message, and one other message, with a total size of 160 bits + 256 bits + 1024 bits + 80 bits = 1529 bits. The patient A sends signature information, transaction information, key information, a hash, and two other pieces of information, totaling 1024 bits + 160 bits + 256 bits + 2 × 80 bits = 1760 bits. The doctor then accesses the IPFS through the hash address to obtain the EMR information to decrypt with the private key, the total size of an EMR, and a key of 1024 bits + 160 bits = 1184 bits. The total communication cost for the data access phase is 1529 bits + 1760 bits + 1184 bits = 4473 bits. As a result, as shown in Table 3, speed is required in various network environments.

### 5.2. Computation Cost

The cost of each phase of the plan for this paper is determined and is displayed in the following Table 4. The analytical basis for estimating the cost is the asymmetric encryption and decryption operations of ECDSA, the hashing operation, the generation of the re-encryption key, and the re-encryption key for proxy re-encryption.

### 5.3. Performance Analysis

**Figure 9:**
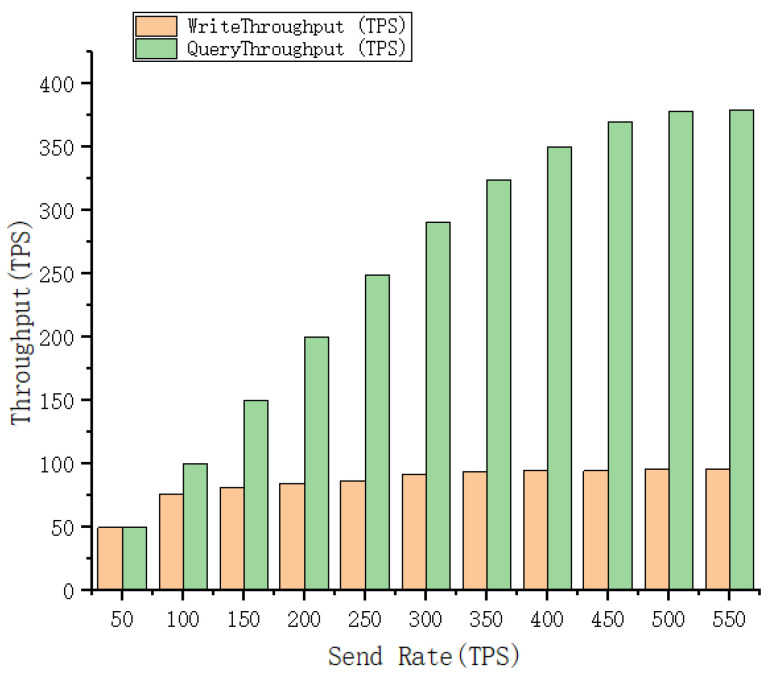
Throughput of Fabric with the varying workload.

**Figure 10:**
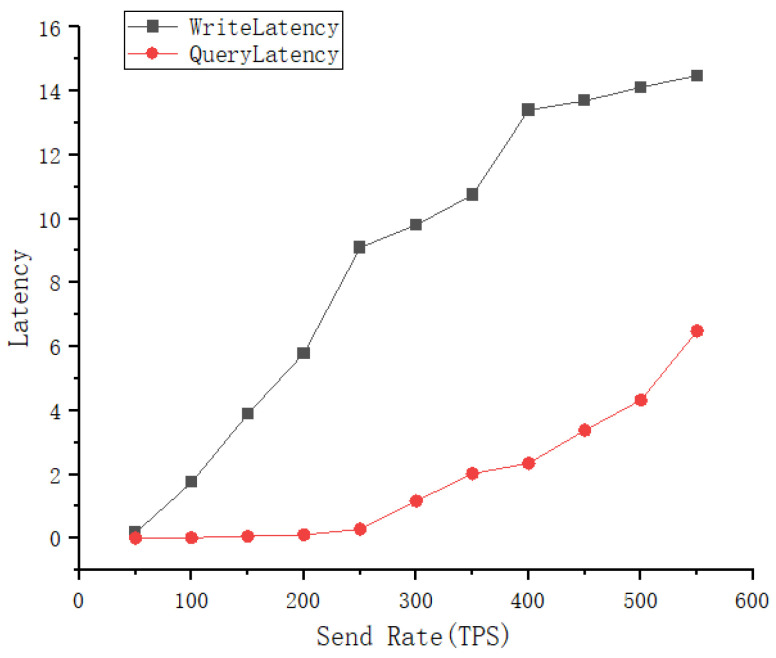
Latency of Fabric with the varying workload.

For the suggested scheme in this study, a performance evaluation of chaincode contract calls is carried out in this section. The testing tool uses Hyperledger Caliper version 0.42 and the blockchain platform uses Hyperledger Fabric version 2.3. Led by the Linux Foundation, it was jointly established by 30 initial corporate members including IBM. On a server with an AMD R7 5800H@3.2GHz CPU and 4GB RAM, we set up one CA node, one order node, and seven peer nodes. Ubuntu 20.04 serves as the machine’s operating system. We have written the core chaincode main operation for the proposed solution for submitting transaction data to the blockchain network and reading data from the network. For this purpose, the throughput and transaction latency of the chaincode were tested using Caliper v0.42 as a performance metric. Throughput in blockchain networks is the rate of transaction submission to the ledger measured in TPS, and latency is the time required to test the interaction between the handover chaincode and the ledger [31]. The transaction latency and throughput for read–write operations are shown in Figure 9 and Figure 10.

In Figure 9, we analyze the connection between send rate and throughput. For this test, 11 groups of send rates, each with a 50-interval range, were chosen. The throughput rises as the send rate rises under the assumption that the block size remains constant. The write transaction’s throughput has a minimum of 49.6 tps, a maximum of 95.6 tps, and a threshold of roughly 95 tps. The minimum and maximum throughput for reading transactions are 50.2 and 379 tps, respectively, with around 380 tps serving as the threshold. In Figure 10, we analyze the connection between send rate and latency. When the block size remains constant, latency increases in tandem with the send rate. The minimum latency for writing data is 0.18 s and the maximum is 14.48 s. At the same time, the latency of query transactions is more efficient before the send rate is 400, and the query speed starts to decrease slightly after reaching the threshold. Therefore, the proposed system has good performance in storing and reading the transaction information of EMR, can quickly obtain the EMR of a patient during his or her visit, and has a large enough throughput to write EMR information after the visit.

### 5.4. Comparison

Table 5 compares the previous approach solution, and it can be inferred that the current solution resolves the issues with the prior solution. Xia et al. [18] suggested a blockchain-based data sharing framework, but it does not fully specify the signature algorithm process, and the access control module lacks flexibility in the custom access policy model section. Wang et al. [19] presented a decentralized approach for access control based on secret policy attribute encryption (CP-ABE) paired with an Ethernet public chain, but it increases the storage on the chain burden and the data owner’s burden. Wholesome Coin, created by Alsalamah et al. [20], is issued using blockchain technology. However, this step is vulnerable to malicious stealing of coin numbers and coin misuse by dishonest people. Even though Mani et al. [21] advocated patient-centric EMR administration using IPFS as a medium to store medical data, there is still a risk of data leakage due to poor data security mechanisms in IPFS. Kan et al. [22] suggested a proxy re-encryption strategy with CAA-secure, which overcomes the problem of decentralized storage security. However, no storage performance tests were performed.

Compared to previous studies, our proposed solution mainly makes it possible to use medical tokens and share EMR securely. All aspects of information confidentiality, non-repudiation, integrity, and data transmission rate are guaranteed. This study utilizes cryptography to ensure the security of the information during transmission.

### 5.5. Architecture Comparison

Public, private, and federated blockchains are now the three main forms of blockchain architecture available on the market [32]. Private blockchain data concentration is not favorable for inter-enterprise data transmission; public blockchain data transparency, which anyone can observe, is not ideal for the storage of confidential data between organizations. Table 6 summarizes the comparison of the three platforms: Bitcoin, Ethereum, and Hyperledger Fabric. As we can see from the table below, the Hyperledger Fabric platform has more flexible pluggable modules than the Bitcoin and ETH platforms, and can be adapted to a variety of application scenarios, such as the one proposed in this paper. At the same time, Hyperledger Fabric has better performance, higher throughput, data traceability, and non-disclosure features.

## 6. Conclusions

Internet healthcare is moving on a new path thanks to blockchain technology. In this work, we propose a shared EMR system with incentives built on top of IPFS and blockchain technologies. To guarantee data confidentiality, integrity, and non-repudiation across the entire system connection, we employ ECDSA. The analysis of identity authentication also employs BAN logic. In contrast to other research suggestions, this system is more concerned with secure data exchange and the incorporation of medical tokens to enable rewarded sharing of EMRs. The examination of the communication protocols also reveals that the system in this paper has a better process in the communication flow, and we define the system framework and examine the security of many parts of the system using cryptography.

In summary, the EMR sharing system proposed in this paper has the following advantages:

1. The EMR sharing system may combine case data from various healthcare facilities, allowing case data to be exchanged when patients must transfer between hospitals and guaranteeing the availability of cases;
2. Through the internet, patients can access their case information and track the status of their medications at any time;
3. It can significantly increase the effectiveness of doctor consultations and prevent duplicate tests while transferring between hospitals;
4. Patients can receive Medcoin to aid with medical costs by sharing their EMR data, and doing so will also advance public health monitoring;
5. To the greatest extent possible, IPFS storage and a proxy re-encryption technique are utilized for data transport. This increases patient EMR security and privacy.

## Figures and Tables

**Table 2:** Non-repudiation of the proposed scheme.

	Item	Signature Value	Sender	Receiver	Signature Verification
Phase					
**Data storage phase**		$\left(r_{P2},s_{P2}\right)$	Patient	Hyperledger Fabric	$x_{P2}^{\prime }\overset{?}{=}r_{P2}mod\;n$
**Data access phase**		$\left(r_{D1},s_{D1}\right)$	Doctor	Hyperledger Fabric	$x_{D1}^{\prime }\overset{?}{=}r_{D1}mod\;n$
		$\left(r_{P3},s_{P3}\right)$	Patient	Doctor	$x_{P3}^{\prime }\overset{?}{=}r_{P3}mod\;n$
**Diagnosis phase**		$\left(r_{D2},s_{D2}\right)$	Doctor	Hyperledger Fabric	$x_{D2}^{\prime }\overset{?}{=}r_{D2}mod\;n$

**Table 3:** Communication cost.

	Item	Message Length	4G (100 Mbps)	5G (20 Gbps)
Phase				
**Registration** **phase**		960 bits	9.6 μs	0.048 μs
**Data storage** **phase**		2880 bits	29 μs	0.114 μs
**Data access** **phase**		3289 bits	33 μs	0.164 μs
**Diagnosis** **phase**		4473 bits	45 μs	0.224 μs

**Table 4:** Computation cost.

	Party	Patient	Doctor	Hyperledger Fabric
Phase				
**Data storage phase**		$T_{Enc}+2T_{Sig}+2T_{H}$	N/A	$T_{H}+T_{Ver}$
**Data access phase**		$T_{rk}+T_{reEnc}+T_{Sig}$	$T_{Sig}+T_{H}+T_{Dec}$	$T_{H}+T_{Ver}$
**Diagnosis phase**		N/A	$T_{Enc}+T_{H}+T_{Sig}$	$T_{H}+T_{Ver}$

Notes: $T_{Enc}$: encryption operation, $T_{Dec}$: decryption operation, $T_{reEnc}$: re-encryption operation, $T_{rk}$: re-encrypt key generation operation, $T_{Sig}$: signature operation, $T_{H}$: hash function operation, $T_{Ver}$: verify operation.

**Table 5:** Functionality comparison of previous schemes and the proposed scheme.

Authors	Year	Objective	1	2	3	4	5	6	7
**Xia et al.** [18]	2017	Solve the problem of data shareability and control in the cloud through blockchain.	Y	Y	Y	N	Y	N	N
**Wang et al.** [19]	2019	Access control of data in cloud storage is achieved through Ethernet combined with secret attribute policy-based encryption.	Y	Y	Y	Y	Y	N	N
**Alsalamah et al.** [20]	2021	Use IoT devices to monitor people’s physical health data, issue medical currencies through blockchain technology, and create a reward mechanism between the two.	Y	N	Y	N	Y	N	Y
**Mani et al.** [21]	2021	Using IPFS to store medical data to solve the blockchain storage capacity problem.	Y	N	Y	N	Y	Y	N
**Kan et al.** [22]	2022	Reduce the cost of re-encryption key replication in a decentralized storage environment with CAA-secure’s proxy re-encryption solution.	Y	Y	Y	N	Y	N	N
**Ours**	2022	Propose a Fabric-based system with an incentive model for sharing and storing medical privacy data.	Y	Y	Y	Y	Y	Y	Y

Notes: 1: blockchain architecture, 2: encryption key, 3: access control, 4: authorization, 5: scalability, 6: off-chain storage, 7: token, (Y): yes, (N): no.

**Table 6:** Comparison between Ethereum and Hyperledger Fabric.

Comparison Items	Bitcoin	Ethereum	Hyperledger Fabric
Description	Quantitative blockchain platform	Modular blockchain platform	Generic blockchain platform
Scene	Public chain	Public chain	Federation chain
Consensus algorithm	Proof of work (POW)	Proof of work (PoW)	Practical byzantine fault tolerance (PBFT)
Throughput	7 TPS	25 TPS	1000 TPS
Decentralization	Complete decentralization	Complete decentralization	Partial de-centralization
Smart contract	No	Yes	Yes
Scalability	No	No	Yes
Authentication	No	No	Yes
Privacy	No	No	Yes
Pluggability	No	No	Yes

## Data Availability

The data used to support the findings of this study are available from the corresponding author upon request.

## Notations Glossary

$ID_{X}$	The identity of *X*
$d_{X}$	The ECDSA private key of the party *X*
$Q_{X}$	The ECDSA public key of the party *X*
$Cert_{X}$	A digital certificate of user *X*
$k_{i}$	The *i*th random number chosen by the user
data	Medical record data
$M_{Pi}$	Collection of encrypted EMR and patient information
$\left(r_{Xi},s_{Xi}\right)$	The *i*th signature generated by user *X*
$H_{Xi}$	The *i*th hash generated by user *X*
$T_{X}$	Role X stores the transaction information in the chain
$Req_{X}$	Request information of role *X*
$rk_{A\to B}$	The re-encryption key authorized by the data holder to the applicant
$\left(C_{X1},C_{X2}\right)$	User *X* ciphertext after encryption with encryption key
$\left(C_{X1}^{\prime },C_{X2}^{\prime }\right)$	User *X* ciphertext after encryption with re-encryption key

## Appendix A

**Algorithm A1.** Encrypted algorithm.
**func Encrypted** (data string, k string) (ciphertext string) {
$\begin{array}{l} \;\;\;\;data\leftarrow Input()\\ \;\;\;\;k\leftarrow Input()\\ \;\;\;\;publickey_{p}\leftarrow patient_{c}ert.getPublicKey\\ \;\;\;\;P_{data}=f(data)\\ \;\;\;\;cipheretext=(k*publickey_{p},z^{k}+P_{d}ata)\\ \;\;\;\;(c1,c2)\leftarrow ciphertext\\ \;\;\;\;return(c1,c2) \end{array}$
}

**Algorithm A2.** ECDSA signature.
**func Sign** (k string, d string, H string) (r string, s string){ $\begin{array}{l} \left(x,y\right)=k*G\\ \;\;\;\;\;\;\;\;r=x/n\\ \;\;\;\;\;\;\;\;if\left(r!=0\right)\{\\ \;\;\;\;\;\;\;\;\;\;\;\;\;s=\left(H+r*d\right)/k\%n\\ \;\;\;\;\;\;\;\;\}else\{\\ \;\;\;\;\;\;\;\;\;\;\;\;\;\;\;\;\;\;return\;false\\ \;\;\;\;\;\;\;\;\}\\ return\;r,s \end{array}$
}

**Algorithm A3.** ECDSA verification.
**func Verify** (H string, r string, s string) (res string){
$\begin{array}{l} \;\;\;\;\;\;\;\;u1=\left(H\;mod\;n\right)/s\\ \;\;\;\;\;\;\;\;u2=\left(r\;mod\;n\right)/s\\ \;\;\;\;\;\;\;\;Q\leftarrow patient\_cert.getPublicKey\\ \;\;\;\;\;\;\;\;\left(x,y\right)=u1*G+u2*Q\\ \begin{array}{l} \;\;\;\;\;\;\;\;if\left(x==r\right)\{\\ \;\;\;\;\;\;\;\;\;\;\;return\;true\}\\ \;\;\;\;\;\;\;\;else\{\\ \;\;\;\;\;\;\;\;return\;false\} \end{array} \end{array}$
}

**Algorithm A4.** Insert a custom attribute in a digital certificate.
*public class RegisterUserWithAttribute {* *RegistrationRequest registrationRequest = new RegistrationRequest(newUser);* *registrationRequest.setAffiliation(“org.Hospital.XXXHospital”);* *registrationRequest.setEnrollmentID(“userID1”);* *registrationRequest.addAttribute(new Attribute(“ROLE”, “H”));//user-defined attr* *String enrollmentSecret = caClient.register(registrationRequest, admin);* *EnrollmentRequest enrollmentRequest = new EnrollmentRequest();* *enrollmentRequest.addAttrReq(“hf.Affiliation”);* *enrollmentRequest.addAttrReq(“hf.EnrollmentID”);* *enrollmentRequest.addAttrReq(“hf.Type”);* *enrollmentRequest.addAttrReq(“ROLE”);* *Enrollment enrollment = caClient.enroll(“userID1”, enrollmentSecret, enrollmentRequest);* *Identity user = Identity.createIdentity(“OrgHMSP”, enrollment.getCert(), enrollment.getKey());* *wallet.put(“userID1”, user);* *System.out.println(“Successfully enrolled user and imported it into the System”);}}*

**Algorithm A5.** Definition of the access control policy followed by the JSON format.
*rule EMRCAndUPermission {* *description: “Only doctor and patient can add and update EHR data, the researcher only can read”* *participant(m): “User”* *operation: READ, UPDATE* *resource(v): “TRANSACTION”* *subject: ((m.Role == “D” && v.Did == m.ID) || (v.Pid == m.ID))* *environment: (BeginTime,EndTime)* *action: ALLOW* *}*

**Algorithm A6.** Re-encryption algorithm.
*func reEncrypted (*ciphertext*string,*rk*string,* e*string,*r*string) (*reciphertext *string){* $\begin{array}{l} \;\;\;\;privatekey_{p}\leftarrow -1/patient\_cert.getPrivateKey\\ \;\;\;\;publickey_{p}\leftarrow patient\_cert.getPublicKey\\ \;\;\;\;publickey_{d}\leftarrow doctor\_cert.getPublicKey\\ \;\;\;\;C_{1}^{\prime }=e*(r*publickey_{p},rk)\\ \;\;\;\;C_{2}^{\prime }=z^{r}*G+P_{data}\\ \;\;\;\;reciphertext\leftarrow (C_{1}^{\prime },C_{2}^{\prime })\\ \;\;\;\;return\;reciphertext\} \end{array}$

**Algorithm A7.** Decryption algorithm.
*func Decrypted (*reciphertext*string) (data string){* $\begin{array}{l} \;\;\;\;P\_data=C_{2}^{\prime }-{\left(C_{1}^{\prime }\right)}^{(1/privatekey_{d})*G}\\ \;\;\;\;data=f^{-1}(P\_\mathrm{data}) \end{array}$ *Return data}*

**Algorithm A8.** Token generation.
*type Token struct {* * Owner string ‘json:”Owner”‘* * TotalSupply unit ‘json:”TotalSupply”‘* * TokenName string ‘json:”TokenName”‘* * TokenSymbol string ‘json:”TokenSymbol”‘* * BalanceOf map[string]uint ‘json:”BalanceOf”‘}* *func (token *Token) initialSupply(){* * token.BalanceOf[token.Owner] = token.TotalSupply;}* *func (token *Token) transfer(_from string, _to string, _value uint){* * if(token.BalanceOf[_from] >= _value){* * token.BalanceOf[_from] -= _value;* * token.BalanceOf[_to] += _value;}}* *func (token *Token) balance(_from string) uint{* * return token.BalanceOf[_from]}* *func (s *SmartContract) InitLedger(ctx contractapi.TransactionContextInterface) error {* * token: =&Token{* * Owner: “medcoin”,* * TotalSupply: 100000000,* * TokenName: “MEDCOIN”,* * TokenSymbol: “MEDC”,* * BalanceOf: map[string]uint{}}* * token.initialSupply()*
